# TMEM106B Knockdown Exhibits a Neuroprotective Effect in Parkinson’s Disease via Decreasing Inflammation and Iron Deposition

**DOI:** 10.1007/s12035-024-04373-4

**Published:** 2024-07-23

**Authors:** Yumei Liu, Kunpeng Qin, Chunyan Jiang, Jinzhao Gao, Binghui Hou, Anmu Xie

**Affiliations:** Department of Neurology, Affiliated Hospital of Qingdao University, No. 16 Jiangsu Road, Qingdao, 266000 Shandong China

**Keywords:** Parkinson’s disease, TMEM106B, Inflammation, Iron deposition

## Abstract

Parkinson’s disease (PD) is closely related to iron accumulation and inflammation. Emerging evidence indicates that TMEM106B plays an essential role in PD. But whether TMEM106B could act on neuroinflammation and iron metabolism in PD has not yet been investigated. The aim of this study was to investigate the pathological mechanisms of inflammation and iron metabolism of TMEM106B in PD. 1-methyl-4-phenylpyridinium (MPP^+^)- and 1-methyl-4-phenyl-1,2,3,6-tetrahydropyridine (MPTP)-induced SH-SY5Y cells and mice were treated with LV-shTMEM106B and AAV-shTMEM106B to construct PD cellular and mouse models. Pole tests and open-field test (OFT) were performed to evaluate the locomotion of the mice. Immunohistochemistry and iron staining were used to detect TH expression and iron deposition in the SN. Iron staining was used to measure the levels of iron. Western blotting was used to detect the expression of inflammatory factors (tumor necrosis factor-α (TNF-α), interleukin-6 (IL-6)), NOD-like receptor protein 3 (NLRP3) inflammasome, divalent metal transporter 1 (DMT1), and Ferroportin1 (FPN1)). Knockdown of TMEM106B improved motor ability and rescued dopaminergic (DA) neuron loss. TMEM106B knockdown attenuated the increases of TNF-α, IL-6, NLRP3 inflammasome, and DMT1 expression in the MPP^+^ and MPTP-induced PD models. Furthermore, TMEM106B knockdown also increases the expression of FPN1. This study provides the first evidence that knockdown of TMEM106B prevents dopaminergic neurodegeneration by modulating neuroinflammation and iron metabolism.

## Introduction

Parkinson’s disease (PD) is the second most common neurodegenerative disease, affecting approximately 2990 per 100,000 individuals over the age of 70 globally [1]. PD is characterized by the abnormal aggregation of α-synuclein (α-syn) deposition and the progressive loss of dopaminergic (DA) neurons in the substantia nigra pars compacta (SNpc), resulting in bradykinesia, resting tremor, myotonia, and other clinical manifestations. At present, the pathogenesis of PD remains uncertain, and there is no effective treatment to delay PD progression.

Emerging studies have shown that dysregulation and accumulation of iron in the SN may contribute to the degeneration of neurons in PD [2, 3]. Dysregulation of iron levels is a crucial factor in the pathogenesis of PD, because it is strongly associated with multiple pathological processes such as oxidative stress, mitochondrial dysfunction, toxic protein aggregation, and iron poisoning [4–8]. Iron levels in the SNpc were found to be significantly elevated compared to age-matched healthy controls [9–12]. The injection of iron within the SN in rats resulted in a decrease in dopaminergic activity, which was mitigated by antioxidant therapy [13, 14]. Recent studies have reported that neuroinflammation is also a characteristic feature of PD pathology [15]. Clinical evidence has suggested that the levels of the proinflammatory cytokines interleukin-6 (IL-6), interleukin-1β (IL-1β), and tumor necrosis factor-α (TNF-α) were higher in the serum and cerebrospinal fluid of PD patients [16, 17]. Indeed, dysregulation of the microglial NOD-like receptor protein 3 (NLRP3) inflammasome caused by microglial activation is thought to be involved in the pathogenesis of PD [18–20]. NLRP3 deletion significantly ameliorated motor impairments and decreased the loss of DA neurons in mice treated with mitochondrial neurotoxin 1-methyl-4-phenyl-1,2,3,6-tetrahydropyridine (MPTP) [21].

Transmembrane protein 106B (TMEM106B) encodes a lysosomal membrane protein and was identified as a risk factor for frontotemporal lobar degeneration (FTLD) [22–25]. Recent studies have indicated that TMEM106B regulates several other neurodegenerative diseases, including PD, Alzheimer’s disease (AD), chronic traumatic encephalopathy (CTE), and limbic-predominant age-related TDP-43 encephalopathy (LATE). In addition, TMEM106B form amyloid fibrils were found in human brains in an age-dependent manner [26–29]. For PD dementia (PDD) patients, the fibrils formed by TMEM106B were also found in the postmortem brain [27]. These findings suggest that TMEM106B play the critical role in PD.

In the central nervous system (CNS), TMEM106B is mainly expressed in neurons and oligodendrocytes [30–33]. TMEM106B deficiency contributes to the impairment of lysosomal trafficking in the axons of motor neurons and Purkinje cells [32, 34] and decreased the survival of Purkinje cells during aging [35–37]. Furthermore, increasing studies have suggested that there is a close association between TMEM106B and inflammation. TMEM106B has been shown to regulate inflammatory polarization of innate immune cells, inflammatory pathways of CNS, and disease-independent degenerative changes [38]. The inflammatory genes were upregulated in TMEM106b knockout mice, suggesting that astrocytes and microglia may be activated [39]. Recent research on transcriptions also exhibited a tight link between TMEM106B and inflammation [38, 40]. But TMEM106B knockout was found to reduce the proliferation and activation of microglia and upregulate microglial apoptosis in mouse models [41]. Nevertheless, there is a lack of research examining the impact of iron accumulation and neuroinflammation in cellular and animal models of PD.

Previous scientists found that young drug users exposed to the MPTP appeared to have PD characteristics [42]. MPTP could be converted to 1-methyl-4-phenylpyridinium (MPP^+^) by the type B monoamine oxidase, thereby producing excess ROS and increasing oxidative stress, and its toxicity could damage mitochondrial respiration via inhibiting complex I [43, 44]. Treatment of the human neuroblastoma SH-SY5Y cell line with MPP^+^ has also become a common in vitro model for elucidating the mechanism of nigra degeneration and therapeutic trials [45]. TMEM106B is mainly expressed in neurons, and previous studies have constructed PD models with MPP^+^[46–49] and MPTP [50, 51] in vitro and in vivo to study PD-related inflammation and iron deposition. Therefore, we treated SH-SY5Y cells and mice with MPP^+^ and MPTP to construct PD models, respectively. Then, we knocked down TMEM106B expression to research the regulatory relationship between TMEM106B and iron accumulation and neuroinflammation in PD. We discovered that TMEM106B deficiency attenuated the accumulation of iron and neuroinflammation.

## Materials and Methods

### Cell Culture

The rigorous protocol employed in the present study was reviewed and approved by the Ethics Committee of the Affiliated Hospital of Qingdao University (QYFY WZLL 28618). Human neuroblastoma cell line SH-SY5Y was purchased from the Chinese Academy of Sciences Cell Bank. Cells were maintained in Dulbecco’s modified Eagle medium (DMEM) supplemented with 10% fetal bovine serum and 1% penicillin–streptomycin a constant temperature incubator at 37 °C, 5% CO_2_.

### Cell Transfection and Treatment

As shown in previous studies [21, 52, 53], SH-SY5Y cells were treated with 1 mM MPP^+^ concentration for 24 h to construct PD cellular model. To decrease the expression of TMEM106B, lentivirus TMEM106B (LV-shTMEM106B) and shRNA control (LV-shNC) were used to transfect SH-SY5Y cells, respectively. The infection efficiency was verified by monitoring the fluorescence of green fluorescent protein (GFP). Then, stable SH-SY5Y cells were obtained by screening with puromycin (8 μg/mL). The knockdown of TMEM106B protein was confirmed by immunoblot analysis.

### Evaluation of ROS by Flow Cytometry

Intracellular reactive oxygen species (ROS) levels were determined using dichlorodihydrofluorescein (DCFH-DA) fluorescence assay. Briefly, SH-SY5Y cells were incubated with 1 mL DCFH-DA (5ul, dissolved in HBS) for 30 min at 37 °C in dark. Thereafter, the cells were rinsed thrice with HBS and resuspended with 1-mL HBS. The ROS levels were analyzed using flow cytometry and expressed as values relative to the control.

### Animals and Treatment

C57BL/6 male mice were purchased from Charles River Laboratories (Beijing, China). All mice were housed in plastic cages alone, with a 12/12-h light/dark cycle, and were given free access to water and food. The housing room temperature was kept at 20–26 °C with a humidity level between 40 and 70%. The mice were adapted to the laboratory environment for at least 1 week before the experiment.

A mouse model of PD was established by intraperitoneal injection of C57BL/6 male mice (8–10 weeks old) with MPTP (30 mg/kg, Sigma) for 5 consecutive days. The same volume of normal saline was intraperitoneally injected into the control and AAV-shNC groups. Mice were deeply anesthetized with isoflurane (3–4% for induction and 2% for maintenance with 0.5% oxygen). Two hundred nanoliters of AAV-shTMEM106B and AAV-shNC was stereotactically microinjected into bilateral SN, respectively (anteroposterior (AP) − 3.0 mm and mediolateral (ML) ± 1.5 mm relative to Bregma and dorsoventral (DV) − 4.5 mm relative to skull surface) according to the mouse brain atlas. In addition, the same volume of 0.9% sterile saline was microinjected into bilateral SN in the control and PD groups. Three weeks after virus microinjection, MPTP was intraperitoneally injected into mice to construct a PD model. C57BL/6 male mice were randomly divided into six groups, and each group contained 15 mice.

### Pole Test

The pole test was used to measure the degree of bradykinesia of mice. Briefly, mice were placed head up on a 0.5-cm diameter ball located at the top of a vertical thick pole, and the time it took them to turn completely (turning time) and the total time it took them to reach the floor were recorded. Each animal was subjected to three consecutive trials, each 5 min apart. The average of three trials was calculated for statistical analysis.

### Open Field Experiment

The open field test is an excellent approach for assessing the overall manifestation of motor impairments in a mouse model of PD. In this experiment, the open field was made out of a 50 × 50-cm plaza box and a 40-cm high fence. Mice were placed in the box in a fixed position and given a few minutes to adjust to their new surroundings. Then, their behavior was captured on tape for around 5 min. The wide area was thoroughly cleaned with 70% alcohol and dried between each mouse.

### Iron Staining

The brain slices were first fixed with 4% paraformaldehyde (PFA) for 5 min, washed by double-distilled water (ddH2O) for 30 s, then incubated with 2% hydrochloric acid and 2% potassium ferrocyanide for 30 min. After washing two 0.01 M PBS for 5 min, slices were incubated with 1% H_2_O_2_ dissolved in methyl alcohol for 20 min to inactivate endogenous catalases. After washing two 0.01 M PBS times in PBS, sections were stained using a DAB chromogenic kit for 15 min.

### Immunofluorescent

The mice were anesthetized with sodium pentobarbital and sacrificed, perfused with normal saline. The brains were dissected and fixed with 4% PFA, and then stored in 20% and 30% sucrose. Then, frozen brains were sectioned coronally at 30 μm. For immunofluorescence staining, brain slices were incubated with an anti-TH antibody (1:500) to detect the level of DA neuron damage. Then, slices were incubated with goat anti-rabbit IgG (Alexa Fluor 594) secondary antibodies, and images were captured using a fluorescence microscope.

### Immunohistochemistry

Brain slices are obtained as described above. After washing three times in PBS, sections were incubated in 10% goat serum for 60 min at room temperature and incubated overnight at 4 °C in anti-TH antibody (1:5000). Then, the sections were incubated with secondary IgG-horseradish peroxidase (HRP), followed by visualization with DAB peroxidase substrate, and photographed by an Olympus microscope. We selected 20 successive sections from each brain for examination. Three mice from each group were used for this measurement.

### Western Blotting Analysis

SH-SY5Y cells and mouse SN were soaked in RIPA buffer and placed on ice. The lysate was centrifuged, the supernatant was collected, and the total protein concentration was determined by bicinchoninic acid (BCA) method. Protein samples were electrophoretic on an SDS gel and transferred to a polyvinylidene fluoride (PVDF) membrane. Subsequently, the membranes were incubated at room temperature in TBST (Tween-20) containing 5% skim milk for 2 h, and then incubated overnight with primary antibody. Then, the membrane was cleaned with TBST for three times, 10 min each time, and incubated with secondary antibody at room temperature for 1 h. After TBST washing for three times and 10 min, enhanced chemiluminescence detection kit was used to detect protein expression levels. Image J software was used to analyze the band.

### Statistical Analysis

All results were analyzed using the GraphPad Prism program. The data is shown as the mean ± standard error of the mean (SEM) and was derived from a minimum of three studies. The statistical significance was measured by two-way ANOVA with Tukey’s post hoc test (multiple groups) or unpaired two-tailed Student’s *t* test (two groups). The statistical difference was considered significant at *P < 0.05, **P < 0.005, ***P < 0.001, and ****P < 0.0001.

## Results

### TMEM106B Expressions was Elevated in SH-SY5Y Cells Exposed to MPP^+^ and the SN of Mice Treated with MPTP

**Fig. 1:**
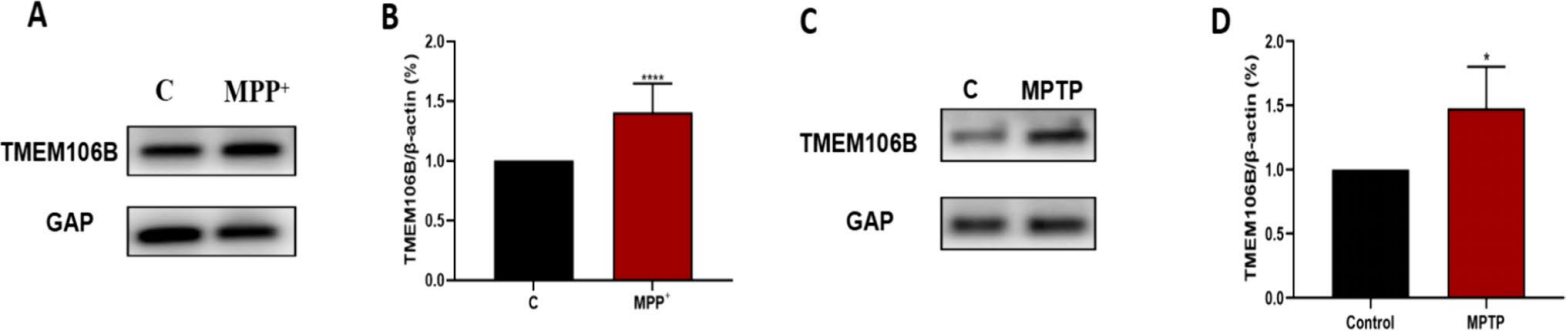
TMEM106B expression is enhanced in PD cellular and mouse models. **A** TMEM106B levels in MPP^+^-treated SH-SY5Y cells were analyzed by western blot analysis. Representative blots are shown. **B** The expression of TMEM106B in MPP^+^-treated SH-SY5Y cells was quantified as the mean ± SEM from three independent experiments. **C** TMEM106B levels in brain SN of MPTP-treated and control mice were analyzed by western blot analysis. Representative blots are shown. **D** The expression of TMEM106B in brain SN of MPTP-treated and control mice was quantified as the mean ± SEM from three independent experiments. Data were analyzed using unpaired two-tailed Student’s *t* test. *P < 0.05; ****P < 0.0001, vs. control group

In order to investigate alterations in TMEM106B expression, we first assessed the expression of TMEM106B in SH-SY5Y cells induced by MPP^+^ and the SN of mice treated with MPTP. Western blot analysis revealed that the levels of TMEM106B were higher after MPP^+^ and MPTP treatment compared with untreated controls (Fig. 1A, B, C, D).

**Fig. 2:**
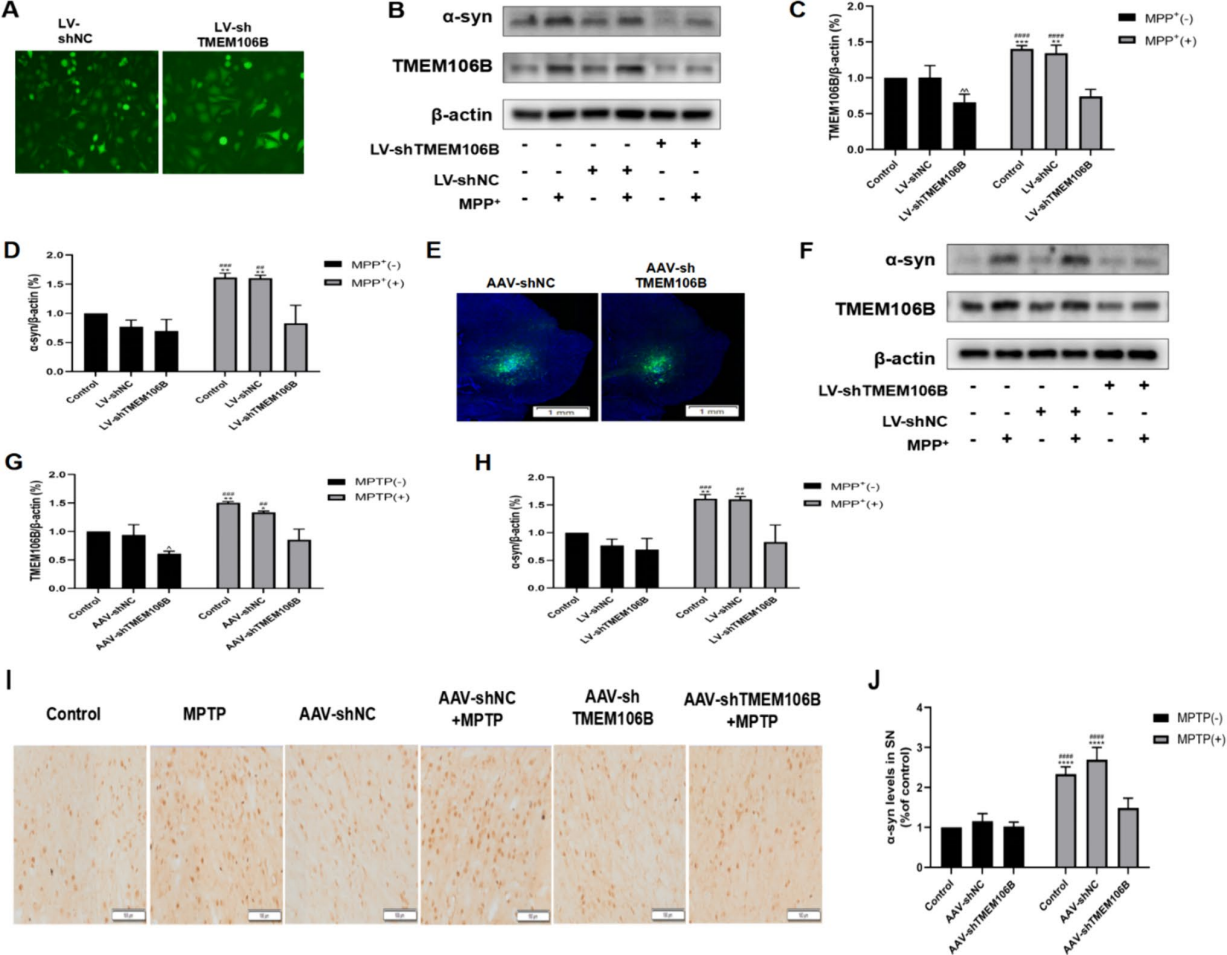
The expression of TMEM106B protein in PD cellular and mouse models. **A** The green fluorescence of the selected cells infected with the LV-shNC and LV-shTMEM106B. **B** The protein levels of TMEM106B and α-syn in the SH-SY5Y cells from different treatment groups were detected by western blot analysis. Representative blots are shown. The levels of TMEM106B (**C**) and α-syn (**D**) in the SH-SY5Y cells from different treatment groups were quantified as the mean ± SEM from three independent experiments. **E** Immunofluorescence of AAV-shNC and AAV-shTMEM106B (green-labeled) in the SN. **F** The protein levels of TMEM106B and α-syn in the SN from different treatment groups were detected by western blot analysis. The expressions of TMEM106B (**G**) and α-syn (**H**) in the SN from different treatment groups were quantified as the mean ± SEM from three independent experiments. **I** Immunohistochemical staining of α-syn levels in the SN from different treatment groups. **J** Data are representative of the results of three independent experiments (mean ± SEM). *P < 0.05, **P < 0.005, ***P < 0.001, ****P < 0.0001, *vs*. control group; ^##^*P* < 0.005, ^###^*P* < 0.001, ^####^*P* < 0.0001, *vs*. LV-shTMEM106B + MPP^+^( +) and AAV-shTMEM106B + MPTP group; ^*P* < 0.05, ^^*P* < 0.005 *vs*. LV-shNC + MPP^+^ and AAV-shNC + MPTP group. Data were analyzed using two-way ANOVA, followed by Tukey’s post hoc test

To decrease the expression of TMEM106B, Lv-shTMEM106B and AAV-shTMEM106B were transferred to SH-SY5Y cells and mice. The Lv-shTMEM106B and AAV-shTMEM106B encoded the green fluorescent protein gene, allowing for the visualization of lentiviral and AAV expression (Fig. 2A, E). In SH-SY5Y cells, TMEM106B expression was significantly reduced in the Lv-shTMEM106B group compared with the Lv-shNC group, suggesting that the lentivirus administration reduced shTMEM106B expression. In addition, the levels of TMEM106B and α-syn were elevated in the MPP^+^ and LV-shNC + MPP^+^ groups compared with the control group. The Lv-shTMEM106B + MPP^+^ group showed lower levels of TMEM106B and α-syn compared with the MPP^+^ and Lv-shNC + MPP^+^ groups (Fig. 2B, C, D). In the SN of mice treated by MPTP, the results were consistent with the results of in vitro experiment (Fig. 2F, G, H). The immunohistochemical staining of α-syn also found similar results with the results of in vivo experiment (Fig. 2I, J). Therefore, the levels of TMEM106B and α-syn were elevated in both PD cellular and mouse models, and TMEM106B knockdown decreased the expression of α-syn. In addition, treatment with Lv-shTMEM106B and AAV-shTMEM106B could effectively inhibit the expression of TMEM106B.

### Inhibition of TMEM106B Expression Attenuated MPP^+^*-*Induced Inflammatory Response and NLRP3 Inflammasome Activation in SH-SY5Y Cells

**Fig. 3:**
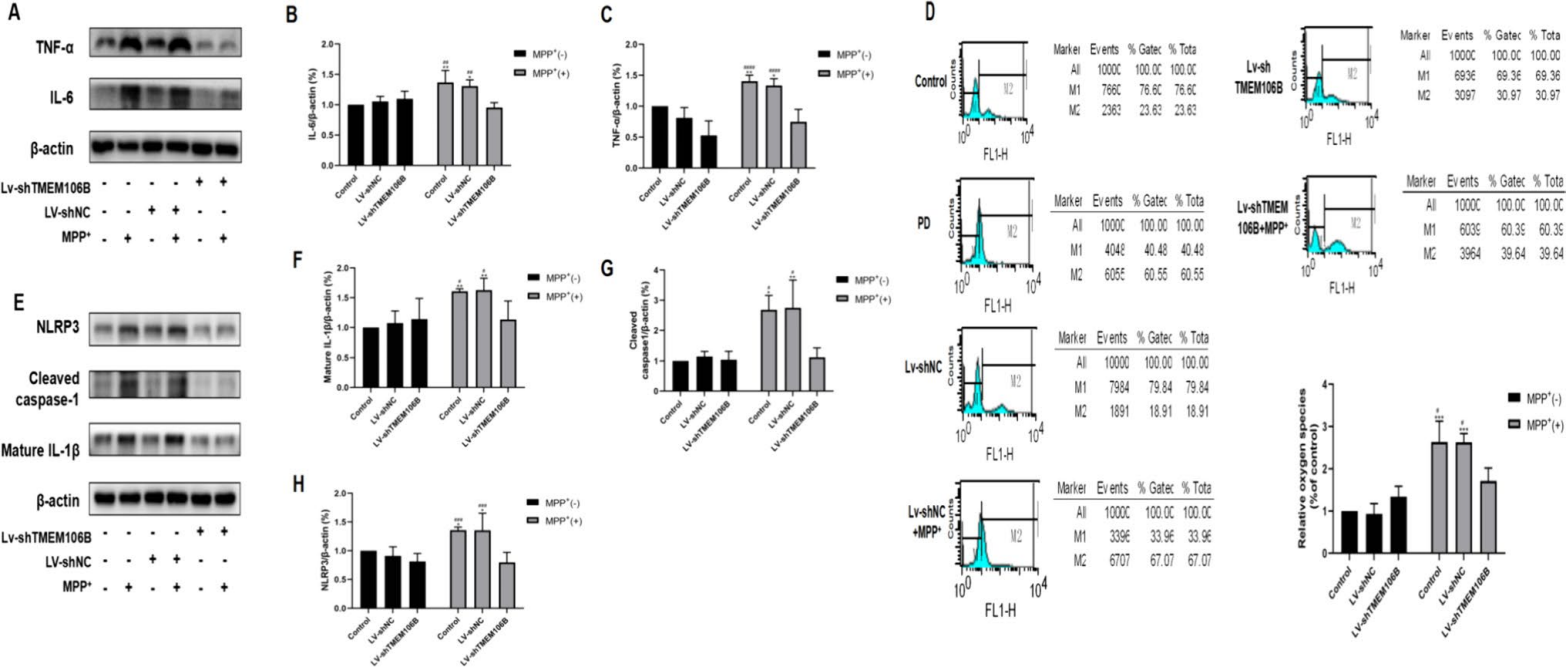
Knockdown of TMEM106B attenuated MPP^+^-induced inflammatory response and NLRP3 inflammasome activation in SH-SY5Y cells. **A** The levels of IL-6 and TNF-α in the SH-SY5Y cells from different treatment groups were determined by western blot. Representative blots are shown. The levels of IL-6 (**B**) and TNF-α (**C**) in the SH-SY5Y cells from different treatment groups were quantified as the mean ± SEM from three independent experiments. **D** The level of ROS was determined by flow cytometry. **E** The levels of mature IL-1β, cleaved caspase-1, and NLRP3 in the SH-SY5Y cells from different treatment groups were determined by western blot. Representative blots are shown. The expressions of mature IL-1β (**F**), cleaved caspase-1 (**G**), and NLRP3 (**H**) in the SH-SY5Y cells from different treatment groups were quantified as the mean ± SEM from three independent experiments. *P < 0.05, **P < 0.005, ***P < 0.001, *vs*. control group; ^#^*P* < 0.05, ^##^*P* < 0.005, ^###^*P* < 0.001, ^####^*P* < 0.0001, *vs*. LV-shTMEM106B + MPP^+^ group. Data were analyzed using two-way ANOVA, followed by Tukey’s post hoc test

It is widely recognized that inflammatory results in the loss of DA neurons. To detect the influence of TMEM106B on inflammation in SH-SY5Y cells induced by MPP^+^, we measured the related proteins by western blot. As shown in Fig. 3A–C, the expression of several proinflammatory factors, including interleukin-6 (IL-6) and TNF-α, was significantly increased in MPP^+^-treated SH-SY5Y cells. Furthermore, compared with the Lv-shNC + MPP^+^ and MPP^+^ groups, the IL-6 and TNF-α expressions were reduced in the Lv-shTMEM106B + MPP^+^ group, and inhibition of TMEM106B effectively decreased cytoplasmic ROS to comparable basal levels, suggesting that inhibition of TMEM106B attenuated the inflammatory response (Fig. 3D).

We also research the influence of TMEM106B on NLRP3 inflammasome activity. As shown in Fig. 3E–H, the NLRP3 inflammasome protein was significantly increased in MPP^+^-treated SH-SY5Y cells. In addition, the protein level of the NLRP3 inflammasome was significantly decreased in Lv-shTMEM106B-treated SH-ST5Y cells following MPP^+^ exposure. Consistent with the change trend of NLRP3 level, the NLRP3-dependent maturation of caspase-1 and production of mature IL-1β exhibited the same results, indicating TMEM106B knockdown could inhibit NLRP3 activation.

### Inhibition of TMEM106B Expression Attenuated Iron Accumulation in SH-SY5Y Cells Treated by MPP^+^

**Fig. 4:**
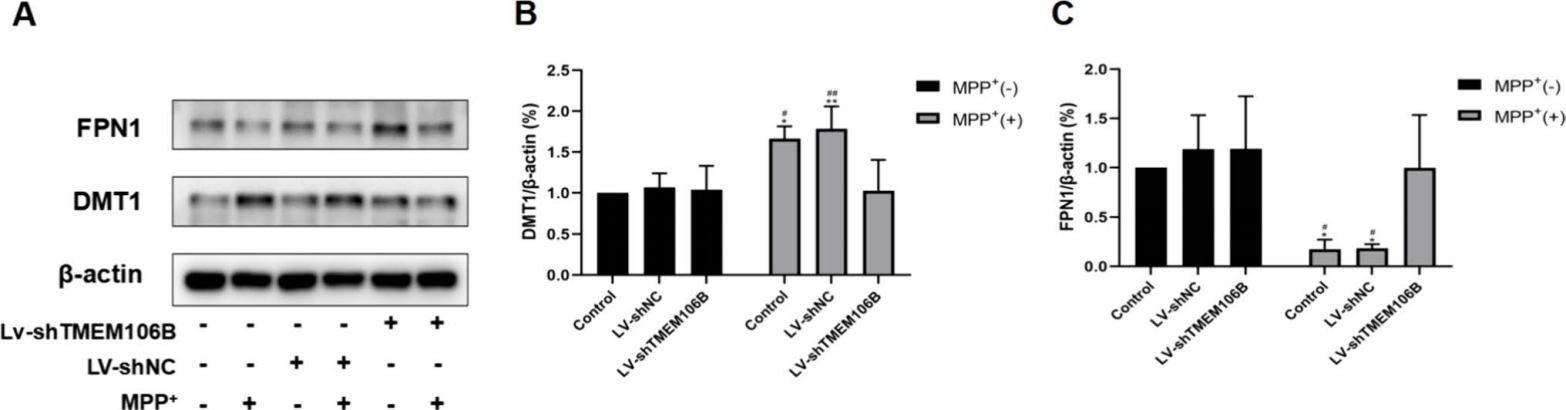
Knockdown of TMEM106B attenuated attenuates iron accumulation in SH-SY5Y cells induced by MPP^+^. **A** The levels of DMT1 and FPN1 in the SH-SY5Y cells from different treatment groups were determined by western blot. Representative blots are shown. The levels of DMT1 (**B**) and FPN1 (**C**) in the SH-SY5Y cells from different treatment groups were quantified as the mean ± SEM from three independent experiments. *P < 0.05, **P < 0.005, *vs*. control group; ^#^*P* < 0.05, ^##^*P* < 0.005, *vs*. LV-shTMEM106B + MPP^+^ group. Data were analyzed using two-way ANOVA, followed by Tukey’s post hoc test

To research the potential influence of TMEM106B on iron homeostasis, the levels of DMT1 and Ferroportin1 (FPN1) proteins were evaluated via western blot analysis. As shown in Fig. 4, the DMT1 expression was increased and FPN1 expression was decreased in SH-SY5Y cells treated with MPP^+^. Furthermore, inhibition of TMEM106B downregulated the DMT1 expression and upregulated the FPN1 expression in SH-SY5Y cells treated with MPP^+^. These results suggested that MPP^+^ could lead to an increase in iron accumulation, whereas inhibition of the level of TMEM106B decreased iron accumulation.

### Inhibition of TMEM106B Ameliorated MPTP-Induced Motor Impairment and the Loss of Dopaminergic Neuron in Mice

MPTP was used to construct PD models in vivo. To assess the influence of TMEM106B on motor impairment in mice treated with MPTP, pole tests and open-field tests were assessed.

**Fig. 5:**
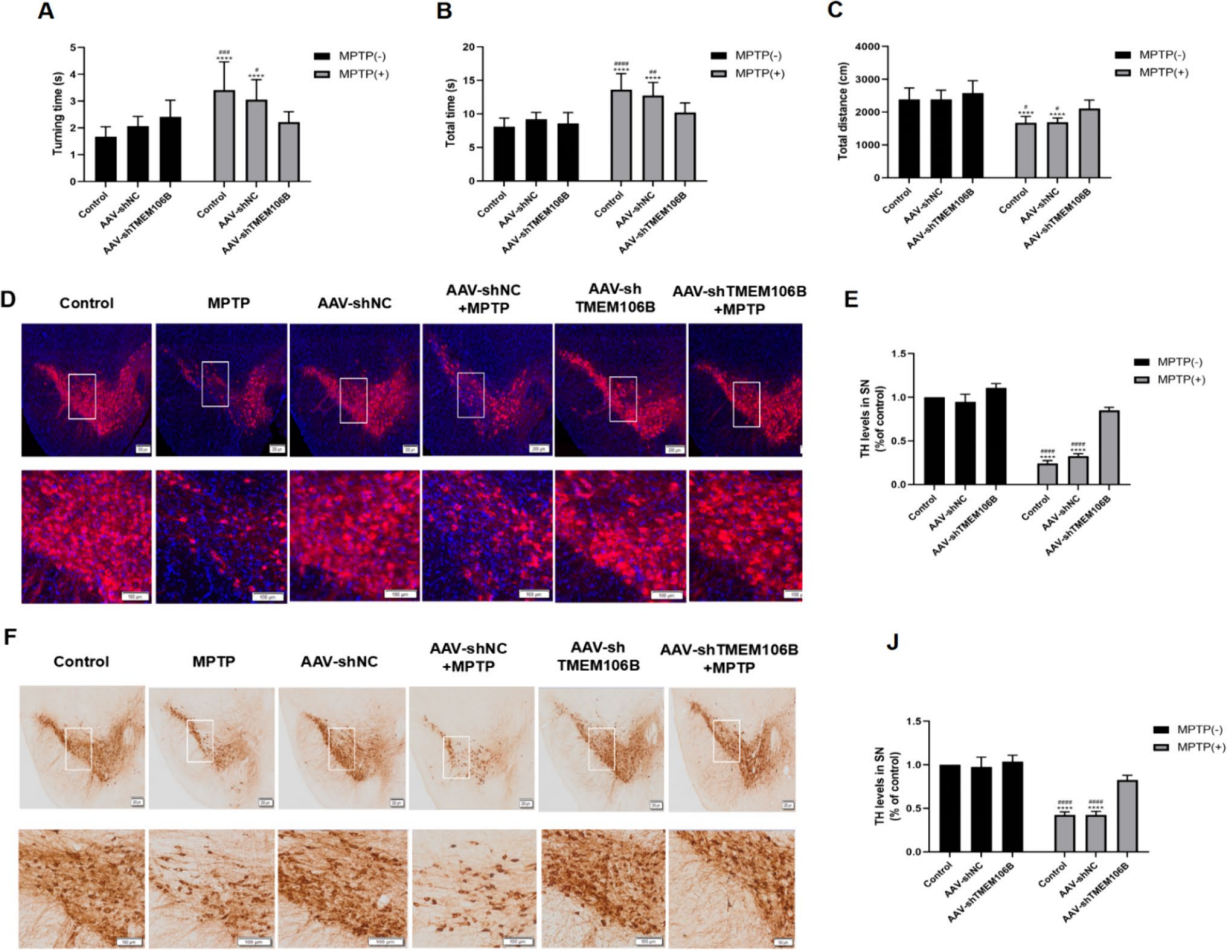
Knockdown of TMEM106B improved motor deficits and attenuated loss of dopaminergic neuron induced by MPTP in mice. In pole test, quantification of the turning (**A**) and total time (**B**) in the pole test for each treatment (mean ± SEM). **C** Open-field test. Quantification of the total distance traveled in the open-field test for each treatment (mean ± SEM). **D** Immunofluorescence of TH-positive neurons in the SN from different treatment groups. **E** Data are representative of the results of three independent experiments (mean ± SEM). **F** Immunohistochemical staining of TH-positive neurons in the SN from different treatment groups. **G** Data are representative of the results of three independent experiments (mean ± SEM). ****P < 0.0001, *vs*. control group; ^#^*P* < 0.05, ^##^*P* < 0.005, ^###^*P* < 0.001, ^####^*P* < 0.0001, *vs*. AAV-shTMEM106B + MPTP group. Data were analyzed using two-way ANOVA, followed by Tukey’s post hoc test

The pole test showed that mice in the MPTP-treated group had significantly longer turning time and total time on the pole compared to the control group. In addition, the AAV-shTMEM106B + MPTP group displayed a significant reduction in turning and total time on the pole compared with those in the AAV-shNC + MPTP group and MPTP group (Fig. 5A, B). The results of the open-field test are presented in Fig. 5C; MPTP treatment significantly increased the total distance compared with the control. The AAV-shTMEM106B + MPTP group showed a significant decrease in total distance compared with the AAV-shNC + MPTP and MPTP groups, indicating inhibition of TMEM106B ameliorated the motor impairment of mice induced by MPTP.

In this study, we used a stereotactic injection technique to inject mice with AAV-shTMEM106B to reduce the expression of TMEM106B in the SN of mice, followed by intraperitoneal injection of MPTP to establish a mouse model of PD. As shown in Fig. 5D–J, the number of TH-positive cells in the SN of mice was significantly reduced in MPTP and AAV-shNC + MPTP compared with the control group. The MPTP-induced PD mouse model was successfully established. Indeed, the number of TH-positive cells in AAV-shTMEM106B + MPTP group was higher compared with those in the AAV-shNC + MPTP and MPTP groups, suggesting that inhibition of TMEM106B could rescue dopaminergic neuron loss.

### Inhibition of TMEM106B Decreased MPTP-Induced Inflammatory Response and NLRP3 Inflammasome Activation in the SN of Mice

**Fig. 6:**
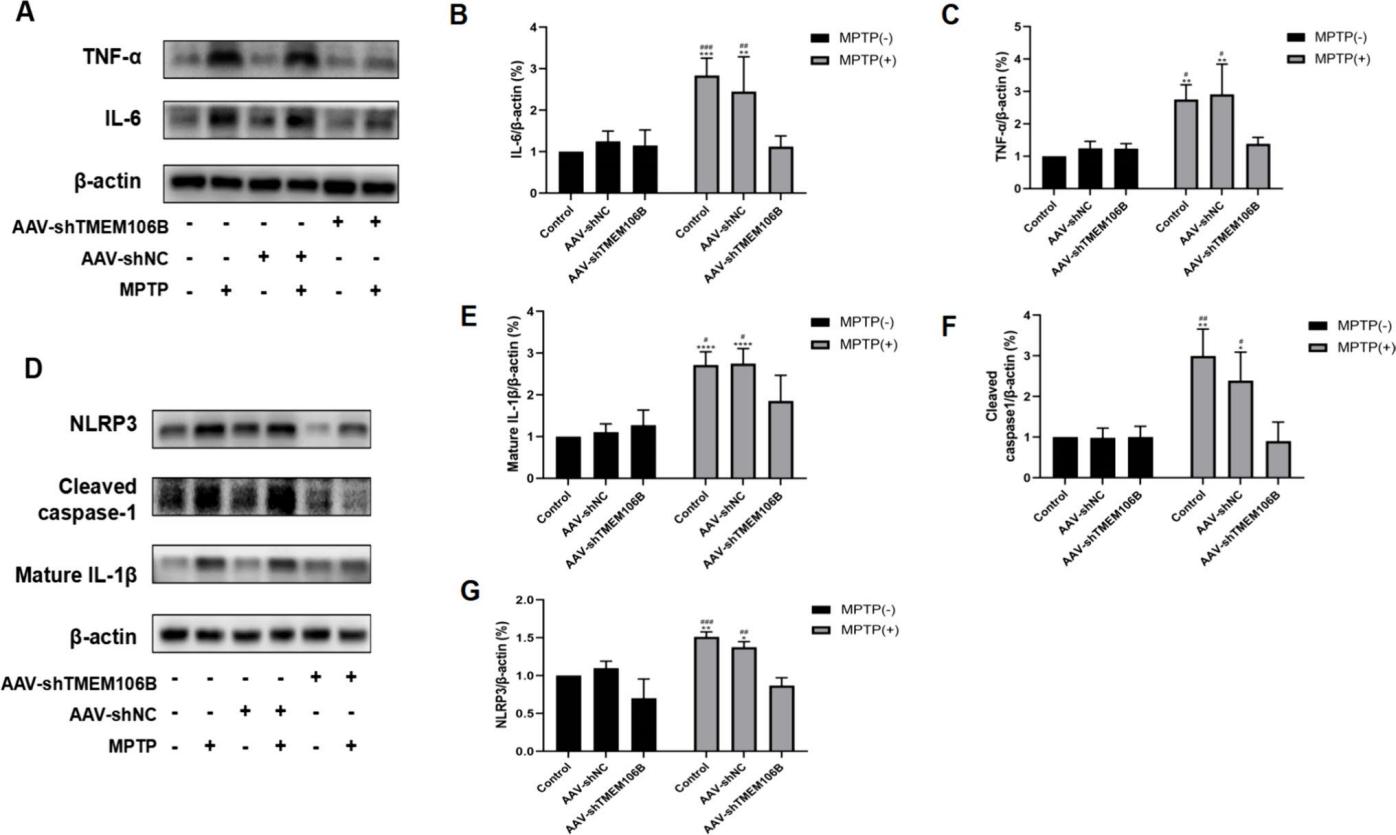
Knockdown of TMEM106B attenuated MPTP-induced inflammatory response and NLRP3 inflammasome activation in SN of mice. **A** The levels of IL-6 and TNF-α in the SN of mice from different treatment groups were determined by western blot. Representative blots are shown. The levels of IL-6 (**B**) and TNF-α (**C**) in the SN of mice from different treatment groups were quantified as the mean ± SEM from three independent experiments. **D** The levels of mature IL-1β, cleaved caspase-1, and NLRP3 in the SN of mice from different treatment groups were determined by western blot. Representative blots are shown. The expressions of mature IL-1β (**E**), cleaved caspase-1 (**F**), and NLRP3 (**G**) in the SN of mice from different treatment groups were quantified as the mean ± SEM from three independent experiments. *P < 0.05, **P < 0.005, ***P < 0.001, ****P < 0.0001, *vs*. control group; ^#^*P* < 0.05, ^##^*P* < 0.005, ^###^*P* < 0.001, *vs*. AAV-shTMEM106B + MPTP group. Data were analyzed using two-way ANOVA, followed by Tukey’s post hoc test

As shown in Fig. 6A–C, consistent with the results of in vitro experiment, IL-6 and TNF-α expressions were upregulated in the MPTP group compared with the control group, indicating that MPTP treatment increased inflammation. Furthermore, compared with the Lv-shNC + MPTP and MPTP groups, the levels of IL-6 and TNF-α were reduced in the AAV-shTMEM106B + MPTP group, showing that inhibition of TMEM106B ameliorated the MPTP-induced inflammatory response.

As shown in Fig. 6D–G. the NLRP3 inflammasome protein was significantly increased in the MPTP group. In addition, the protein level of NLRP3 inflammasome was significantly decreased in the AAV-shTMEM106B + MPTP compared with the AAV-shNC + MPTP and MPTP groups. The cleaved caspase-1 and production of mature IL-1β exhibited the same results, indicating TMEM106B knockdown could inhibit NLRP3 activation.

### Inhibition of TMEM106B Ameliorated MPTP-Induced Iron Accumulation in the SN of Mice

**Fig. 7:**
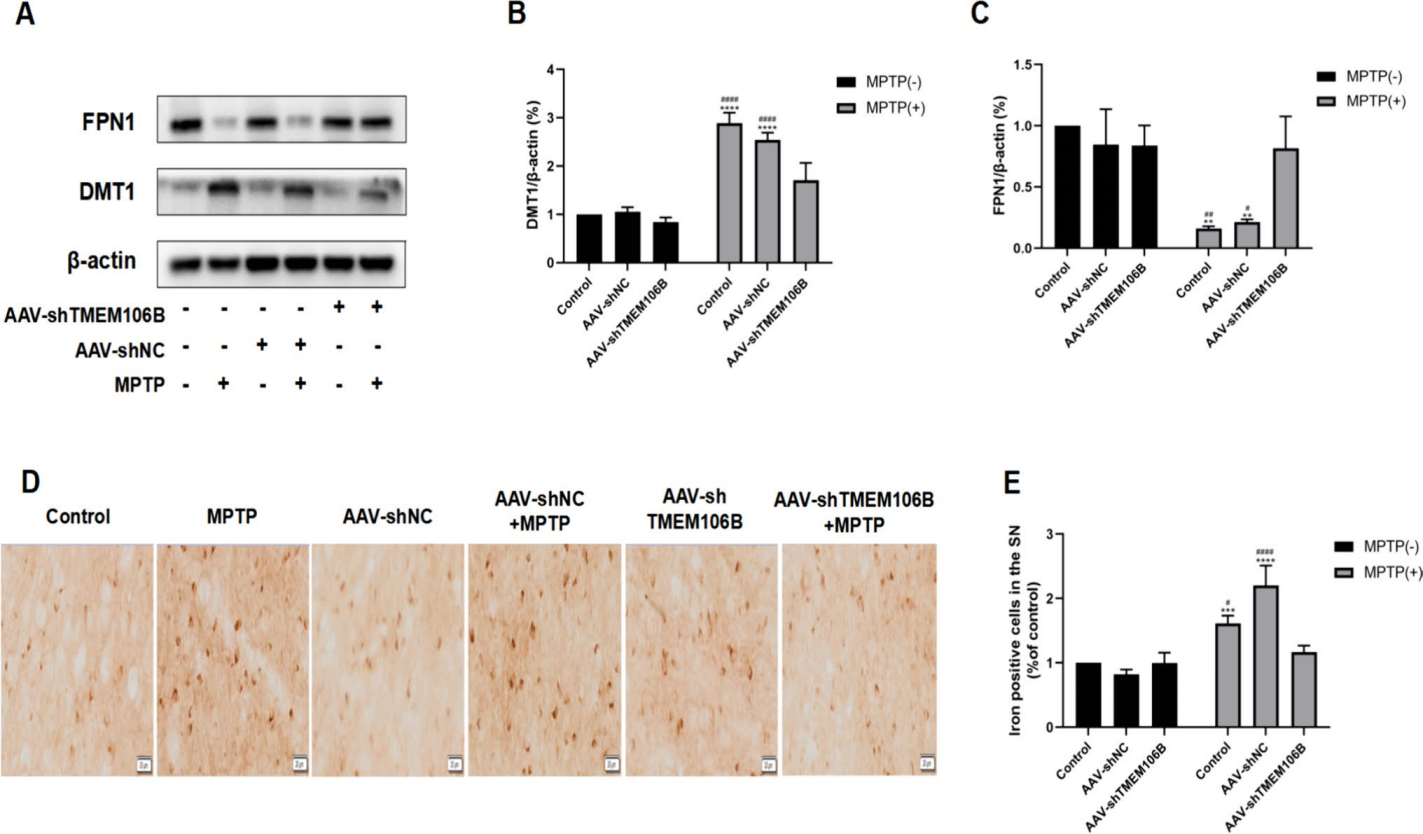
Knockdown of TMEM106B attenuated attenuates iron accumulation in SN of mice treated with MPTP. **A** The levels of DMT1 and FPN1 in SN of mice from different treatment groups were determined by western blot. Representative blots are shown. The levels of DMT1 (**B**) and FPN1 (**C**) in SN of mice from different treatment groups were quantified as the mean ± SEM from three independent experiments. **D** The levels of iron were determined by iron staining. **E** Data are presented as the mean ± SEM from three independent experiments. **P < 0.005, ***P < 0.001, ****P < 0.0001, *vs*. control group; ^#^*P* < 0.05, ^##^*P* < 0.005, ^####^*P* < 0.0001, *vs*. AAV-shTMEM106B + MPTP group. Data were analyzed using two-way ANOVA, followed by Tukey’s post hoc test

In accordance with the findings of an in vitro experiment, the DMT1 expression was elevated while FPN1 expression was reduced in mice treated with MPTP compared with the control group. Compared with the AAV-shNC + MPTP and MPTP groups, the DMT1 level in AAV-shTMEM106B + MPTP group was decreased, and the FPN1 level was increased, indicating that AAV-shTMEM106B treatment significantly attenuated the MPTP-induced increase in iron level (Fig. 7A–C). In addition, MPTP treatment significantly increased iron-positive–stained cells in the SN compared to controls, revealing that MPTP treatment increased iron accumulation. TMEM106B knockdown attenuated MPTP and AAV-shNC + MPTP-induced elevated iron levels (Fig. 7D, E). The above results demonstrated that inhibition of TMEM106B ameliorated the iron accumulation in the mice treated with MPTP.

## Discussion

Our study shows that TMEM106B plays a crucial function in PD. First, the TMEM106B expression was increased in SH-SY5Y cells induced by MPP^+^ and in SN of mice treated with MPTP. Second, inhibition of TMEM106B decreased the DA neuron loss in the SN, improving motor impairment in MPTP-induced PD mice. Thirdly, we observed that inhibition of TMEM106B decreased the expression of inflammatory factors and attenuated neuroinflammation in SH-SY5Y cells induced by MPP^+^ and in the SN of mice treated with MPTP. Finally, inhibition of the expression of TMEM106B ameliorated iron accumulation in PD models. The study highlights that inhibition of TMEM106B expression may represent a potential therapeutic target for PD.

Emerging studies support that neuroinflammation is an important pathology and marker of the progression of PD [54, 55]. Neuroinflammation in specific regions of the brain is caused by a proinflammatory cytokine response and an increase in toxic free radicals [56, 57]. In the SN of postmortem human samples, the expression levels of IL-1β, IL-6, TNF-α, iNOS, and ROS were significantly elevated, indicating the activation of microglia and astrocytes [58, 59]. Neuroinflammation increases PD progression by worsening the loss of DA neurons [60, 61]. It is reported that the inflammatory factors were released by microglial cells due to MPTP treatment, resulting in dopaminergic neuronal death and behavioral impairments [57, 62]. In addition, clinical studies have reported that nonsteroidal anti-inflammatory drugs (NSAIDs) might decrease the risk of PD, showing the important role of neuroinflammation in PD [63–65]. Consistent with previous studies, our research also found that IL-6, TNF-α, and mature IL-1β were elevated in PD cellular and animal models, suggesting that neuroinflammation was upregulated in PD. Furthermore, inhibition of the expression of TMEM106B reduced the levels of IL-6, TNF-α, and mature IL-1β, suggesting that the neuroprotective effect of inhibition of TMEM106B may be related to the suppression of neuroinflammation.

Inflammasome-associated neuroinflammation is a continuing process in PD. The inflammasome is a multiprotein complex that acts as a sensor for the intracellular environment and cellular stress [66]. The NLRP3 inflammasome consists of NLRP3, procaspase-1, and apoptosis-associated spot-like proteins containing caspase recruitment domain proteins [67]. The NLRP3 inflammasome recruits the precursor form of caspase-1, resulting in the cleavage of caspase-1, which is responsible for the maturation and secretion of IL-1β and IL-18. These cytokines are considered to be the cause of neurodegeneration [68, 69]. The studies have reported that the NLRP3 inflammasome, caspase-1, IL-1β, and IL-18 were upregulated in PD patients and PD animal models [18]. In the brains of PD patients, oxidative and the abnormal aggregation of a-syn could activate the inflammasome pathway [70], and the NLRP3 inflammasome protein was increased in in microglia located in the SN of PD patients [70, 71]. Inhibition of NLRP3 decreased inflammasome activation, improved motor impairments, and decreased the abnormal accumulation of α-syn in PD models [21]. Our research also reported that the NLRP3 inflammasome, cleaved caspase-1, and mature IL-1β were increased in PD cellular and animal models. Inhibition of TMEM106B suppressed the NLRP3 inflammasome, cleaved caspase-1, and mature IL-1β expression in SH-SY5Y cells induced by MPP^+^ and SN in mice treated by MPTP.

Iron is an essential nutrient for almost all biological organisms and is widely involved in physiological and biochemical functions [72–74]. Increasing studies reported that the iron accumulation was elevated in the SNpc of PD patients compared to age-matched healthy controls [10–12, 75], as well as the rapid accumulation of iron in the SN of animals treated by 6-OHDA and MPTP [76]. The influx, efflux, and storage of iron within the cell maintain iron homeostasis, which involves many proteins. DMT1 is one of the iron transporters involved in the iron-transported way-NTBI pathway [77]. Results from PD patients also suggested that high levels of DMT1 may be responsible for the accumulation of iron in PD [78]. In addition, 6-OHDA-treated astrocytes showed increased DMT1 expression, while enhancing their ability to transport free iron within the cell, thereby preventing pathogenic iron deposition [79]. FPN1, also known as SLC11A3, is a multi-transmembrane domain protein that is present in several cell types and is involved in cellular iron output, with evidence that iron output is solely dependent on FPN1 [80]. Previous findings suggest that 6-OHDA also decreases FPN1 expression, which further contributes to iron accumulation and toxic damage in DA neurons [8, 71, 81–83]. Consistent with previous studies, our research found that the DMT1 expression was increased and FPN1 expression was decreased in PD models, resulting in an increase in iron burden [84]. Inhibition of the expression of TMEM106B downregulated DMT1 expression and upregulated the FPN1 expression, suggesting that inhibition of TMEM106B could suppress iron accumulation induced by MPP^+^ and MPTP.

In this study, we found that inhibition of TMEM106B expression suppressed neuro-inflammation and alleviated iron accumulation in PD cellular and animal models. Emerging evidence shows a potential correlation between inflammation and iron accumulation in PD. When iron is sequestered intracellularly by altering iron fusion on the cell membrane, excess iron deposition in the SNpc of PD may be related to the cellular response of iron homeostasis to an inflammatory response [84]. IL-1β and TNF-α were reported to regulate the expression of DMT1, affecting iron levels in neuronal cells [85, 86]. The release of proinflammatory cytokines upregulated the DMT1 protein level and downregulated the FPN1 protein level, thereby increasing iron accumulation [86–88]. Further study is required to research the interaction of neuroinflammation and iron accumulation while inhibiting TMEM106B expression. Our research was limited to the TMEM016B knockdown model, indicating the necessity of conducting further studies using a TMEM106B overexpression model.

Taken together, this study suggests that the expression of TMEM106B was upregulated in SH-SY5Y cells induced by MPP^+^ and in the SN of mice treated by MPTP. Inhibition of TMEM106B suppressed neuroinflammation and iron accumulation, prevented the loss of DA neuron, and improved the motor deficits of mice induced by MPTP. Therefore, inhibiting the expression of TMEM106B may be a potential treatment for PD.

## Data Availability

No datasets were generated or analysed during the current study.

## References

[1] Pringsheim T, Jette N, Frolkis A, Steeves TD (2014) The prevalence of Parkinson’s disease: a systematic review and meta-analysis. Mov Disord 29(13):1583–1590. 10.1002/mds.25945 24976103 10.1002/mds.25945

[2] Mochizuki H, Choong CJ, Baba K (2020) Parkinson’s disease and iron. J Neural Transm (Vienna) 127(2):181–187. 10.1007/s00702-020-02149-3 32025811 10.1007/s00702-020-02149-3

[3] Bergsland N, Tavazzi E, Schweser F, Jakimovski D, Hagemeier J, Dwyer MG, Zivadinov R (2019) Targeting iron dyshomeostasis for treatment of neurodegenerative disorders. CNS Drugs 33(11):1073–1086. 10.1007/s40263-019-00668-6 31556017 10.1007/s40263-019-00668-6 PMC6854324

[4] Elkouzi A, Vedam-Mai V, Eisinger RS, Okun MS (2019) Emerging therapies in Parkinson disease - repurposed drugs and new approaches. Nat Rev Neurol 15(4):204–223. 10.1038/s41582-019-0155-7 30867588 10.1038/s41582-019-0155-7 PMC7758837

[5] Crichton RR, Ward RJ, Hider RC (2019) The efficacy of iron chelators for removing iron from specific brain regions and the pituitary-ironing out the brain. Pharmaceuticals (Basel) 12 (3). 10.3390/ph12030138 10.3390/ph12030138 PMC6789569 31533229

[6] Nunez MT, Chana-Cuevas P (2018) New perspectives in iron chelation therapy for the treatment of neurodegenerative diseases. Pharmaceuticals (Basel) 11 (4). 10.3390/ph11040109 10.3390/ph11040109 PMC6316457 30347635

[7] Liang T, Qian ZM, Mu MD, Yung WH, Ke Y (2020) Brain hepcidin suppresses major pathologies in experimental Parkinsonism. iScience 23 (7):101284. 10.1016/j.isci.2020.101284 10.1016/j.isci.2020.101284 PMC7334576 32623334

[8] Jiang H, Song N, Xu H, Zhang S, Wang J, Xie J (2010) Up-regulation of divalent metal transporter 1 in 6-hydroxydopamine intoxication is IRE/IRP dependent. Cell Res 20(3):345–356. 10.1038/cr.2010.20 20125122 10.1038/cr.2010.20

[9] Dexter DT, Wells FR, Agid F, Agid Y, Lees AJ, Jenner P, Marsden CD (1987) Increased nigral iron content in postmortem parkinsonian brain. Lancet 2(8569):1219–1220. 10.1016/s0140-6736(87)91361-4 2890848 10.1016/s0140-6736(87)91361-4

[10] Hirsch EC, Brandel JP, Galle P, Javoy-Agid F, Agid Y (1991) Iron and aluminum increase in the substantia nigra of patients with Parkinson’s disease: an X-ray microanalysis. J Neurochem 56(2):446–451. 10.1111/j.1471-4159.1991.tb08170.x 1988548 10.1111/j.1471-4159.1991.tb08170.x

[11] Faucheux BA, Martin ME, Beaumont C, Hauw JJ, Agid Y, Hirsch EC (2003) Neuromelanin associated redox-active iron is increased in the substantia nigra of patients with Parkinson’s disease. J Neurochem 86(5):1142–1148. 10.1046/j.1471-4159.2003.01923.x 12911622 10.1046/j.1471-4159.2003.01923.x

[12] Foley PB, Hare DJ, Double KL (2022) A brief history of brain iron accumulation in Parkinson disease and related disorders. J Neural Transm (Vienna) 129(5–6):505–520. 10.1007/s00702-022-02505-5 35534717 10.1007/s00702-022-02505-5 PMC9188502

[13] Ben-Shachar D, Youdim MB (1991) Intranigral iron injection induces behavioral and biochemical “parkinsonism” in rats. J Neurochem 57(6):2133–2135. 10.1111/j.1471-4159.1991.tb06432.x 1940919 10.1111/j.1471-4159.1991.tb06432.x

[14] Wesemann W, Blaschke S, Solbach M, Grote C, Clement HW, Riederer P (1994) Intranigral injected iron progressively reduces striatal dopamine metabolism. J Neural Transm Park Dis Dement Sect 8(3):209–214. 10.1007/BF02260941 7748464 10.1007/BF02260941

[15] Wang Q, Liu Y, Zhou J (2015) Neuroinflammation in Parkinson’s disease and its potential as therapeutic target. Transl Neurodegener 4:19. 10.1186/s40035-015-0042-0 26464797 10.1186/s40035-015-0042-0 PMC4603346

[16] Dufek M, Hamanova M, Lokaj J, Goldemund D, Rektorova I, Michalkova Z, Sheardova K, Rektor I (2009) Serum inflammatory biomarkers in Parkinson’s disease. Parkinsonism Relat Disord 15(4):318–320. 10.1016/j.parkreldis.2008.05.014 18672391 10.1016/j.parkreldis.2008.05.014

[17] Chen X, Hu Y, Cao Z, Liu Q, Cheng Y (2018) Cerebrospinal fluid inflammatory cytokine aberrations in Alzheimer’s disease, Parkinson’s disease and amyotrophic lateral sclerosis: a systematic review and meta-analysis. Front Immunol 9:2122. 10.3389/fimmu.2018.02122 30283455 10.3389/fimmu.2018.02122 PMC6156158

[18] Haque ME, Akther M, Jakaria M, Kim IS, Azam S, Choi DK (2020) Targeting the microglial NLRP3 inflammasome and its role in Parkinson’s disease. Mov Disord 35(1):20–33. 10.1002/mds.27874 31680318 10.1002/mds.27874

[19] Lunemann JD, Malhotra S, Shinohara ML, Montalban X, Comabella M (2021) Targeting inflammasomes to treat neurological diseases. Ann Neurol 90(2):177–188. 10.1002/ana.26158 34219266 10.1002/ana.26158

[20] de Araujo FM, Cuenca-Bermejo L, Fernandez-Villalba E, Costa SL, Silva VDA, Herrero MT (2022) Role of Microgliosis and NLRP3 inflammasome in Parkinson’s disease pathogenesis and therapy. Cell Mol Neurobiol 42(5):1283–1300. 10.1007/s10571-020-01027-6 33387119 10.1007/s10571-020-01027-6 PMC11421755

[21] Lee E, Hwang I, Park S, Hong S, Hwang B, Cho Y, Son J, Yu JW (2019) MPTP-driven NLRP3 inflammasome activation in microglia plays a central role in dopaminergic neurodegeneration. Cell Death Differ 26(2):213–228. 10.1038/s41418-018-0124-5 29786072 10.1038/s41418-018-0124-5 PMC6329843

[22] Finch N, Carrasquillo MM, Baker M, Rutherford NJ, Coppola G, Dejesus-Hernandez M, Crook R, Hunter T et al (2011) TMEM106B regulates progranulin levels and the penetrance of FTLD in GRN mutation carriers. Neurology 76(5):467–474. 10.1212/WNL.0b013e31820a0e3b 21178100 10.1212/WNL.0b013e31820a0e3b PMC3034409

[23] Van Deerlin VM, Sleiman PM, Martinez-Lage M, Chen-Plotkin A, Wang LS, Graff-Radford NR, Dickson DW, Rademakers R et al (2010) Common variants at 7p21 are associated with frontotemporal lobar degeneration with TDP-43 inclusions. Nat Genet 42(3):234–239. 10.1038/ng.536 20154673 10.1038/ng.536 PMC2828525

[24] van der Zee J, Van Langenhove T, Kleinberger G, Sleegers K, Engelborghs S, Vandenberghe R, Santens P, Van den Broeck M et al (2011) TMEM106B is associated with frontotemporal lobar degeneration in a clinically diagnosed patient cohort. Brain 134(Pt 3):808–815. 10.1093/brain/awr007 21354975 10.1093/brain/awr007 PMC3044834

[25] van Blitterswijk M, Mullen B, Nicholson AM, Bieniek KF, Heckman MG, Baker MC, DeJesus-Hernandez M, Finch NA et al (2014) TMEM106B protects C9ORF72 expansion carriers against frontotemporal dementia. Acta Neuropathol 127(3):397–406. 10.1007/s00401-013-1240-4 24385136 10.1007/s00401-013-1240-4 PMC3944829

[26] Chang A, Xiang X, Wang J, Lee C, Arakhamia T, Simjanoska M, Wang C, Carlomagno Y et al (2022) Homotypic fibrillization of TMEM106B across diverse neurodegenerative diseases. Cell 185 (8):1346–1355 e1315. 10.1016/j.cell.2022.02.026 10.1016/j.cell.2022.02.026 PMC9018563 35247328

[27] Fan Y, Zhao Q, Xia W, Tao Y, Yu W, Chen M, Liu Y, Zhao J et al (2022) Generic amyloid fibrillation of TMEM106B in patient with Parkinson’s disease dementia and normal elders. Cell Res 32(6):585–588. 10.1038/s41422-022-00665-3 35477998 10.1038/s41422-022-00665-3 PMC9160068

[28] Schweighauser M, Arseni D, Bacioglu M, Huang M, Lovestam S, Shi Y, Yang Y, Zhang W et al (2022) Age-dependent formation of TMEM106B amyloid filaments in human brains. Nature 605(7909):310–314. 10.1038/s41586-022-04650-z 35344985 10.1038/s41586-022-04650-z PMC9095482

[29] Jiang YX, Cao Q, Sawaya MR, Abskharon R, Ge P, DeTure M, Dickson DW, Fu JY et al (2022) Amyloid fibrils in FTLD-TDP are composed of TMEM106B and not TDP-43. Nature 605(7909):304–309. 10.1038/s41586-022-04670-9 35344984 10.1038/s41586-022-04670-9 PMC9844993

[30] Brady OA, Zheng Y, Murphy K, Huang M, Hu F (2013) The frontotemporal lobar degeneration risk factor, TMEM106B, regulates lysosomal morphology and function. Hum Mol Genet 22(4):685–695. 10.1093/hmg/dds475 23136129 10.1093/hmg/dds475 PMC3554197

[31] Chen-Plotkin AS, Unger TL, Gallagher MD, Bill E, Kwong LK, Volpicelli-Daley L, Busch JI, Akle S et al (2012) TMEM106B, the risk gene for frontotemporal dementia, is regulated by the microRNA-132/212 cluster and affects progranulin pathways. J Neurosci 32(33):11213–11227. 10.1523/JNEUROSCI.0521-12.2012 22895706 10.1523/JNEUROSCI.0521-12.2012 PMC3446826

[32] Feng T, Mai S, Roscoe JM, Sheng RR, Ullah M, Zhang J, Katz II, Yu H et al (2020) Loss of TMEM106B and PGRN leads to severe lysosomal abnormalities and neurodegeneration in mice. EMBO Rep 21(10):e50219. 10.15252/embr.202050219 32852886 10.15252/embr.202050219 PMC7534636

[33] Lang CM, Fellerer K, Schwenk BM, Kuhn PH, Kremmer E, Edbauer D, Capell A, Haass C (2012) Membrane orientation and subcellular localization of transmembrane protein 106B (TMEM106B), a major risk factor for frontotemporal lobar degeneration. J Biol Chem 287(23):19355–19365. 10.1074/jbc.M112.365098 22511793 10.1074/jbc.M112.365098 PMC3365973

[34] Luningschror P, Werner G, Stroobants S, Kakuta S, Dombert B, Sinske D, Wanner R, Lullmann-Rauch R et al (2020) The FTLD risk factor TMEM106B regulates the transport of lysosomes at the axon initial segment of motoneurons. Cell Rep 30 (10):3506–3519 e3506. 10.1016/j.celrep.2020.02.060 10.1016/j.celrep.2020.02.060 32160553

[35] Stroobants S, D’Hooge R, Damme M (2021) Aged TMEM106b knockout mice display gait deficits in coincidence with Purkinje cell loss and only limited signs of non-motor dysfunction. Brain Pathol 31(2):223–238. 10.1111/bpa.12903 33016371 10.1111/bpa.12903 PMC8018119

[36] Rademakers R, Nicholson AM, Ren Y, Koga S, Nguyen HP, Brooks M, Qiao W, Quicksall ZS et al (2021) Loss of TMEM106b leads to cerebellum Purkinje cell death and motor deficits. Brain Pathol 31(3):e12945. 10.1111/bpa.12945 33709463 10.1111/bpa.12945 PMC8412084

[37] Feng T, Luan L, Katz II, Ullah M, Van Deerlin VM, Trojanowski JQ, Lee EB, Hu F (2022) TMEM106B deficiency impairs cerebellar myelination and synaptic integrity with Purkinje cell loss. Acta Neuropathol Commun 10(1):33. 10.1186/s40478-022-01334-7 35287730 10.1186/s40478-022-01334-7 PMC8919601

[38] Rhinn H, Abeliovich A (2017) Differential aging analysis in human cerebral cortex identifies variants in TMEM106B and GRN that regulate aging phenotypes. Cell Syst 4 (4):404–415 e405. 10.1016/j.cels.2017.02.009 10.1016/j.cels.2017.02.009 28330615

[39] Feng T, Sheng RR, Sole-Domenech S, Ullah M, Zhou X, Mendoza CS, Enriquez LCM, Katz II et al (2020) A role of the frontotemporal lobar degeneration risk factor TMEM106B in myelination. Brain 143(7):2255–2271. 10.1093/brain/awaa154 32572497 10.1093/brain/awaa154 PMC7363491

[40] Milind N, Preuss C, Haber A, Ananda G, Mukherjee S, John C, Shapley S, Logsdon BA et al (2020) Transcriptomic stratification of late-onset Alzheimer’s cases reveals novel genetic modifiers of disease pathology. PLoS Genet 16(6):e1008775. 10.1371/journal.pgen.1008775 32492070 10.1371/journal.pgen.1008775 PMC7295244

[41] Zhang T, Pang W, Feng T, Guo J, Wu K, Nunez Santos M, Arthanarisami A, Nana AL, et al (2023) TMEM106B regulates microglial proliferation and survival in response to demyelination. Sci Adv 9 (18):eadd2676. 10.1126/sciadv.add2676 10.1126/sciadv.add2676 PMC10162677 37146150

[42] Langston JW, Ballard P, Tetrud JW, Irwin I (1983) Chronic Parkinsonism in humans due to a product of meperidine-analog synthesis. Science 219(4587):979–980. 10.1126/science.6823561 6823561 10.1126/science.6823561

[43] Meredith GE, Rademacher DJ (2011) MPTP mouse models of Parkinson’s disease: an update. J Parkinsons Dis 1(1):19–33. 10.3233/JPD-2011-11023 23275799 10.3233/JPD-2011-11023 PMC3530193

[44] Li T, Zhang W, Kang X, Yang R, Li R, Huang L, Chen J, Yang Q et al (2020) Salidroside protects dopaminergic neurons by regulating the mitochondrial MEF2D-ND6 pathway in the MPTP/MPP(+) -induced model of Parkinson’s disease. J Neurochem 153(2):276–289. 10.1111/jnc.14868 31520529 10.1111/jnc.14868

[45] Shishido T, Nagano Y, Araki M, Kurashige T, Obayashi H, Nakamura T, Takahashi T, Matsumoto M et al (2019) Synphilin-1 has neuroprotective effects on MPP(+)-induced Parkinson’s disease model cells by inhibiting ROS production and apoptosis. Neurosci Lett 690:145–150. 10.1016/j.neulet.2018.10.020 30316984 10.1016/j.neulet.2018.10.020

[46] Yang YL, Lin TK, Huang YH (2022) MiR-29a inhibits MPP + - induced cell death and inflammation in Parkinson’s disease model in vitro by potential targeting of MAVS. Eur J Pharmacol 934:175302. 10.1016/j.ejphar.2022.175302 36174668 10.1016/j.ejphar.2022.175302

[47] Bae JE, Kim JB, Jo DS, Park NY, Kim YH, Lee HJ, Kim SH, Kim SH, et al (2022) Carnitine protects against MPP(+)-induced neurotoxicity and inflammation by promoting primary ciliogenesis in SH-SY5Y cells. Cells 11 (17). 10.3390/cells11172722 10.3390/cells11172722 PMC9454591 36078130

[48] Zhang Z, Hou L, Song JL, Song N, Sun YJ, Lin X, Wang XL, Zhang FZ et al (2014) Pro-inflammatory cytokine-mediated ferroportin down-regulation contributes to the nigral iron accumulation in lipopolysaccharide-induced Parkinsonian models. Neuroscience 257:20–30. 10.1016/j.neuroscience.2013.09.037 24183966 10.1016/j.neuroscience.2013.09.037

[49] Lin X, Mao L, Chen Q, Wang T, Tao T, Pan L (2024) CircHIVEP2 alleviates Parkinson’s nerve damage and inflammatory response by targeting miR-485-3p. Exp Gerontol 188:112387. 10.1016/j.exger.2024.112387 38431178 10.1016/j.exger.2024.112387

[50] Lun P, Ji T, Wan DH, Liu X, Chen XD, Yu S, Sun P (2022) HOTTIP downregulation reduces neuronal damage and microglial activation in Parkinson’s disease cell and mouse models. Neural Regen Res 17(4):887–897. 10.4103/1673-5374.322475 34472490 10.4103/1673-5374.322475 PMC8530116

[51] Han X, Sun S, Sun Y, Song Q, Zhu J, Song N, Chen M, Sun T et al (2019) Small molecule-driven NLRP3 inflammation inhibition via interplay between ubiquitination and autophagy: implications for Parkinson disease. Autophagy 15(11):1860–1881. 10.1080/15548627.2019.1596481 30966861 10.1080/15548627.2019.1596481 PMC6844502

[52] Kim HY, Jeon H, Kim H, Koo S, Kim S (2018) Sophora flavescens Aiton decreases MPP(+)-induced mitochondrial dysfunction in SH-SY5Y cells. Front Aging Neurosci 10:119. 10.3389/fnagi.2018.00119 29740311 10.3389/fnagi.2018.00119 PMC5928137

[53] Song Q, Peng S, Zhu X (2021) Baicalein protects against MPP(+)/MPTP-induced neurotoxicity by ameliorating oxidative stress in SH-SY5Y cells and mouse model of Parkinson’s disease. Neurotoxicology 87:188–194. 10.1016/j.neuro.2021.10.003 34666128 10.1016/j.neuro.2021.10.003

[54] Janda E, Boi L, Carta AR (2018) Microglial phagocytosis and its regulation: a therapeutic target in Parkinson’s disease? Front Mol Neurosci 11:144. 10.3389/fnmol.2018.00144 29755317 10.3389/fnmol.2018.00144 PMC5934476

[55] Yokoyama H, Uchida H, Kuroiwa H, Kasahara J, Araki T (2011) Role of glial cells in neurotoxin-induced animal models of Parkinson’s disease. Neurol Sci 32(1):1–7. 10.1007/s10072-010-0424-0 21107876 10.1007/s10072-010-0424-0

[56] McGeer PL, McGeer EG (2004) Inflammation and neurodegeneration in Parkinson’s disease. Parkinsonism Relat Disord 10(Suppl 1):S3-7. 10.1016/j.parkreldis.2004.01.005 15109580 10.1016/j.parkreldis.2004.01.005

[57] Block ML, Zecca L, Hong JS (2007) Microglia-mediated neurotoxicity: uncovering the molecular mechanisms. Nat Rev Neurosci 8(1):57–69. 10.1038/nrn2038 17180163 10.1038/nrn2038

[58] Ransohoff RM (2016) How neuroinflammation contributes to neurodegeneration. Science 353(6301):777–783. 10.1126/science.aag2590 27540165 10.1126/science.aag2590

[59] Tansey MG, McCoy MK, Frank-Cannon TC (2007) Neuroinflammatory mechanisms in Parkinson’s disease: potential environmental triggers, pathways, and targets for early therapeutic intervention. Exp Neurol 208(1):1–25. 10.1016/j.expneurol.2007.07.004 17720159 10.1016/j.expneurol.2007.07.004 PMC3707134

[60] Shao W, Zhang SZ, Tang M, Zhang XH, Zhou Z, Yin YQ, Zhou QB, Huang YY et al (2013) Suppression of neuroinflammation by astrocytic dopamine D2 receptors via alphaB-crystallin. Nature 494(7435):90–94. 10.1038/nature11748 23242137 10.1038/nature11748

[61] Hirsch EC, Hunot S (2009) Neuroinflammation in Parkinson’s disease: a target for neuroprotection? Lancet Neurol 8(4):382–397. 10.1016/S1474-4422(09)70062-6 19296921 10.1016/S1474-4422(09)70062-6

[62] Kumar H, Kim IS, More SV, Kim BW, Bahk YY, Choi DK (2013) Gastrodin protects apoptotic dopaminergic neurons in a toxin-induced Parkinson’s disease model. Evid Based Complement Alternat Med 2013:514095. 10.1155/2013/514095 23533492 10.1155/2013/514095 PMC3603713

[63] San Luciano M, Tanner CM, Meng C, Marras C, Goldman SM, Lang AE, Tolosa E, Schule B et al (2020) Nonsteroidal anti-inflammatory use and LRRK2 Parkinson’s disease penetrance. Mov Disord 35(10):1755–1764. 10.1002/mds.28189 32662532 10.1002/mds.28189 PMC7572560

[64] Rees K, Stowe R, Patel S, Ives N, Breen K, Clarke CE, Ben-Shlomo Y (2011) Non-steroidal anti-inflammatory drugs as disease-modifying agents for Parkinson’s disease: evidence from observational studies. Cochrane Database Syst Rev (11):CD008454. 10.1002/14651858.CD008454.pub2 10.1002/14651858.CD008454.pub2 PMC13579550 22071848

[65] Gao X, Chen H, Schwarzschild MA, Ascherio A (2011) Use of ibuprofen and risk of Parkinson disease. Neurology 76(10):863–869. 10.1212/WNL.0b013e31820f2d79 21368281 10.1212/WNL.0b013e31820f2d79 PMC3059148

[66] Yan Y, Jiang W, Liu L, Wang X, Ding C, Tian Z, Zhou R (2015) Dopamine controls systemic inflammation through inhibition of NLRP3 inflammasome. Cell 160(1–2):62–73. 10.1016/j.cell.2014.11.047 25594175 10.1016/j.cell.2014.11.047

[67] Walsh JG, Muruve DA, Power C (2014) Inflammasomes in the CNS. Nat Rev Neurosci 15(2):84–97. 10.1038/nrn3638 24399084 10.1038/nrn3638

[68] Martinon F, Burns K, Tschopp J (2002) The inflammasome: a molecular platform triggering activation of inflammatory caspases and processing of proIL-beta. Mol Cell 10(2):417–426. 10.1016/s1097-2765(02)00599-3 12191486 10.1016/s1097-2765(02)00599-3

[69] Ramesh G, MacLean AG, Philipp MT (2013) Cytokines and chemokines at the crossroads of neuroinflammation, neurodegeneration, and neuropathic pain. Mediators Inflamm 2013:480739. 10.1155/2013/480739 23997430 10.1155/2013/480739 PMC3753746

[70] Gordon R, Albornoz EA, Christie DC, Langley MR, Kumar V, Mantovani S, Robertson AAB, Butler MS, et al (2018) Inflammasome inhibition prevents alpha-synuclein pathology and dopaminergic neurodegeneration in mice. Sci Transl Med 10 (465). 10.1126/scitranslmed.aah4066 10.1126/scitranslmed.aah4066 PMC6483075 30381407

[71] Wang S, Yuan YH, Chen NH, Wang HB (2019) The mechanisms of NLRP3 inflammasome/pyroptosis activation and their role in Parkinson’s disease. Int Immunopharmacol 67:458–464. 10.1016/j.intimp.2018.12.019 30594776 10.1016/j.intimp.2018.12.019

[72] de la Fuente-Fernandez R, Calne DB (2002) Evidence for environmental causation of Parkinson’s disease. Parkinsonism Relat Disord 8(4):235–241. 10.1016/s1353-8020(01)00055-4 12039417 10.1016/s1353-8020(01)00055-4

[73] Chen LL, Huang YJ, Cui JT, Song N, Xie J (2019) Iron dysregulation in Parkinson’s disease: focused on the autophagy-lysosome pathway. ACS Chem Neurosci 10(2):863–871. 10.1021/acschemneuro.8b00390 30590010 10.1021/acschemneuro.8b00390

[74] Chen L, Li C, Xie J (2021) Axonal iron transport might contribute to iron deposition in Parkinson’s disease. Neurosci Bull 37(2):275–277. 10.1007/s12264-020-00585-5 32964366 10.1007/s12264-020-00585-5 PMC7870726

[75] Sofic E, Lange KW, Jellinger K, Riederer P (1992) Reduced and oxidized glutathione in the substantia nigra of patients with Parkinson’s disease. Neurosci Lett 142(2):128–130. 10.1016/0304-3940(92)90355-b 1454205 10.1016/0304-3940(92)90355-b

[76] Finkelstein DI, Billings JL, Adlard PA, Ayton S, Sedjahtera A, Masters CL, Wilkins S, Shackleford DM et al (2017) The novel compound PBT434 prevents iron mediated neurodegeneration and alpha-synuclein toxicity in multiple models of Parkinson’s disease. Acta Neuropathol Commun 5(1):53. 10.1186/s40478-017-0456-2 28659169 10.1186/s40478-017-0456-2 PMC5490188

[77] Xu H, Jiang H, Wang J, Xie J (2010) Rg1 protects the MPP+-treated MES23.5 cells via attenuating DMT1 up-regulation and cellular iron uptake. Neuropharmacology 58 (2):488–494. 10.1016/j.neuropharm.2009.09.002 10.1016/j.neuropharm.2009.09.002 19744503

[78] Salazar J, Mena N, Hunot S, Prigent A, Alvarez-Fischer D, Arredondo M, Duyckaerts C, Sazdovitch V et al (2008) Divalent metal transporter 1 (DMT1) contributes to neurodegeneration in animal models of Parkinson’s disease. Proc Natl Acad Sci U S A 105(47):18578–18583. 10.1073/pnas.0804373105 19011085 10.1073/pnas.0804373105 PMC2587621

[79] Zhang HY, Wang ND, Song N, Xu HM, Shi LM, Jiang H, Xie JX (2013) 6-hydroxydopamine promotes iron traffic in primary cultured astrocytes. Biometals 26(5):705–714. 10.1007/s10534-013-9647-x 23771608 10.1007/s10534-013-9647-x

[80] Zhou F, Chen Y, Fan G, Feng C, Du G, Zhu G, Li Y, Jiao H et al (2014) Lead-induced iron overload and attenuated effects of ferroportin 1 overexpression in PC12 cells. Toxicol In Vitro 28(8):1339–1348. 10.1016/j.tiv.2014.07.005 25051259 10.1016/j.tiv.2014.07.005

[81] Song N, Wang J, Jiang H, Xie J (2010) Ferroportin 1 but not hephaestin contributes to iron accumulation in a cell model of Parkinson’s disease. Free Radic Biol Med 48(2):332–341. 10.1016/j.freeradbiomed.2009.11.004 19913091 10.1016/j.freeradbiomed.2009.11.004

[82] Singh N, Haldar S, Tripathi AK, McElwee MK, Horback K, Beserra A (2014) Iron in neurodegenerative disorders of protein misfolding: a case of prion disorders and Parkinson’s disease. Antioxid Redox Signal 21(3):471–484. 10.1089/ars.2014.5874 24512387 10.1089/ars.2014.5874 PMC4076993

[83] Junxia X, Hong J, Wenfang C, Ming Q (2003) Dopamine release rather than content in the caudate putamen is associated with behavioral changes in the iron rat model of Parkinson’s disease. Exp Neurol 182(2):483–489. 10.1016/s0014-4886(03)00123-7 12895460 10.1016/s0014-4886(03)00123-7

[84] Ward RJ, Zucca FA, Duyn JH, Crichton RR, Zecca L (2014) The role of iron in brain ageing and neurodegenerative disorders. Lancet Neurol 13(10):1045–1060. 10.1016/S1474-4422(14)70117-6 25231526 10.1016/S1474-4422(14)70117-6 PMC5672917

[85] Wang J, Song N, Jiang H, Wang J (1832) Xie J (2013) Pro-inflammatory cytokines modulate iron regulatory protein 1 expression and iron transportation through reactive oxygen/nitrogen species production in ventral mesencephalic neurons. Biochim Biophys Acta 5:618–625. 10.1016/j.bbadis.2013.01.021 10.1016/j.bbadis.2013.01.021 23376588

[86] Urrutia P, Aguirre P, Esparza A, Tapia V, Mena NP, Arredondo M, Gonzalez-Billault C, Nunez MT (2013) Inflammation alters the expression of DMT1, FPN1 and hepcidin, and it causes iron accumulation in central nervous system cells. J Neurochem 126(4):541–549. 10.1111/jnc.12244 23506423 10.1111/jnc.12244

[87] Rathnasamy G, Ling EA, Kaur C (2011) Iron and iron regulatory proteins in amoeboid microglial cells are linked to oligodendrocyte death in hypoxic neonatal rat periventricular white matter through production of proinflammatory cytokines and reactive oxygen/nitrogen species. J Neurosci 31(49):17982–17995. 10.1523/JNEUROSCI.2250-11.2011 22159112 10.1523/JNEUROSCI.2250-11.2011 PMC6634148

[88] Thomsen MS, Andersen MV, Christoffersen PR, Jensen MD, Lichota J, Moos T (2015) Neurodegeneration with inflammation is accompanied by accumulation of iron and ferritin in microglia and neurons. Neurobiol Dis 81:108–118. 10.1016/j.nbd.2015.03.013 25801802 10.1016/j.nbd.2015.03.013

